# Genome-wide association study reveals candidate genes influencing lipids and diterpenes contents in *Coffea arabica* L

**DOI:** 10.1038/s41598-017-18800-1

**Published:** 2018-01-11

**Authors:** Gustavo C. Sant’Ana, Luiz F. P. Pereira, David Pot, Suzana T. Ivamoto, Douglas S. Domingues, Rafaelle V. Ferreira, Natalia F. Pagiatto, Bruna S. R. da Silva, Lívia M. Nogueira, Cintia S. G. Kitzberger, Maria B. S. Scholz, Fernanda F. de Oliveira, Gustavo H. Sera, Lilian Padilha, Jean-Pierre Labouisse, Romain Guyot, Pierre Charmetant, Thierry Leroy

**Affiliations:** 1 Instituto Agronômico do Paraná, Laboratório de Biotecnologia Vegetal, 86047902 Londrina, PR Brazil; 2 CIRAD, UMR AGAP, F-34398 Montpellier, France; 3 Empresa Brasileira de Pesquisa Agropecuária, 70770901 Brasília, DF Brazil; 4 Universidade Estadual Paulista, Instituto de Biociências, 13506900 Rio Claro, SP Brazil; 5 IRD, CIRAD, Univ. Montpellier, IPME, BP 64501, 34394 Montpellier, France; 6 AGAP, Univ. Montpellier, CIRAD, INRA, Montpellier SupAgro, Montpellier, France

## Abstract

Lipids, including the diterpenes cafestol and kahweol, are key compounds that contribute to the quality of coffee beverages. We determined total lipid content and cafestol and kahweol concentrations in green beans and genotyped 107 *Coffea arabica* accessions, including wild genotypes from the historical FAO collection from Ethiopia. A genome-wide association study was performed to identify genomic regions associated with lipid, cafestol and kahweol contents and cafestol/kahweol ratio. Using the diploid *Coffea canephora* genome as a reference, we identified 6,696 SNPs. Population structure analyses suggested the presence of two to three groups (K = 2 and K = 3) corresponding to the east and west sides of the Great Rift Valley and an additional group formed by wild accessions collected in western forests. We identified 5 SNPs associated with lipid content, 4 with cafestol, 3 with kahweol and 9 with cafestol/kahweol ratio. Most of these SNPs are located inside or near candidate genes related to metabolic pathways of these chemical compounds in coffee beans. In addition, three trait-associated SNPs showed evidence of directional selection among cultivated and wild coffee accessions. Our results also confirm a great allelic richness in wild accessions from Ethiopia, especially in accessions originating from forests in the west side of the Great Rift Valley.

## Introduction

Coffee beverage popularity is related to its unique aroma and flavor as well as its stimulant properties. The precursors of aroma and flavor, which characterize the beverage, correspond to the chemical compounds of green coffee beans^1^. The concentrations of those components, such as sucrose, caffeine, chlorogenic acids and lipids, are genetically controlled and can be selected to improve beverage quality^2^. Lipids are key compounds involved in flavor and aroma^3^. The coffee lipid fraction is mainly composed of triacylglycerols, sterols, tocopherols and diterpenes. Cafestol (CAF), kahweol (KAH), and 16-O-methyl cafestol are the main diterpenes found in coffee oil^4^. These diterpenes, which are specific to the *Coffea* genus, have both desirable and adverse effects on human health^5,6^. Previous studies of CAF and KAH diterpenes in *Coffea arabica* L. suggested a strong genetic control of their biosynthesis^2,7^. Despite their importance, as far as we know, there is no study trying to correlate the variability of these biochemical compounds among accessions with nucleotide diversity that would be of key interest to optimize coffee breeding strategies.

The southwest Ethiopian highlands are the place of origin of *C*. *arabica*, and several landraces of this species are known from this region^8^. To increase the diversity of *C*. *arabica* breeding programs, research teams have been collecting accessions from various parts of Ethiopia since 1928^9^, transferring germplasm to other tropical countries. One important survey was organized by FAO in 1964–1965, and harvested seeds were sent to India, Tanzania, Ethiopia, Costa Rica, Portugal, and Peru^10^. The Instituto Agronômico do Paraná (IAPAR - Londrina, PR, Brazil) received 132 of those accessions in 1976, which were planted and maintained to this day. The accessions available in this collection show great phenotypic variation in plant architecture, and size of branches, leaves, fruits, and seeds. In relation to biotic and abiotic factors, these coffee accessions exhibit various levels of tolerance and resistance^11,12^. In addition to these morphological and agronomical characteristics, these accessions present a large variability in terms of biochemical contents in green beans, which often translates into a large range of beverage qualities^2,12,13^.

*C*. *arabica* is an allotetraploid (2n = 4 ×  = 44), which is derived from a spontaneous hybridization between two closely related diploid species, *Coffea eugenioides*^14^ and *Coffea canephora* Pierre ex A. Froehner^15^. Whereas *C*. *canephora* (2n = 2 ×  = 22) is an allogamous diploid species harboring a high diversity^16^, the propagation history of *C*. *arabica* combined with its autogamy has led to a narrow genetic diversity among cultivars^17^. *C*. *arabica* breeding programs suffered from this lack of diversity, which also hampered the development of molecular tools whose efficiency is recognized as maximizing the genetic gains per unit of time. Genetic maps have only recently been reported for C. *arabica*^18^. However, there is no publicly available *C*. *arabica* reference genome, even though a few research efforts have been started. Nevertheless, a diploid genomic reference of *C*. *canephora* has been released and has allowed significant progress for *C*. *arabica* genomic analyses^19,20^.

Genome-wide association studies (GWAS) are an efficient approach to dissect the genetic architecture of complex traits^21^. GWAS usually provides a higher mapping-resolution than classical biparental QTL mapping experiments, and is considered as a cost-effective way to detect associations between molecular markers and traits of interest^21,22^. However, assessing the population structure of the association panel is necessary to minimize the occurrence of spurious associations^21^. GWAS requires the use of an adequate number of markers. Recently, next-generation sequencing platforms have dramatically reduced the cost and time to obtain large numbers of markers. Because of its relative simplicity and robustness, the genotyping-by-sequencing (GBS) strategies have been extensively used^21,22^.

In this study, our objectives were to (i) identify SNPs within *C*. *arabica* genotypes based on GBS analyses; (ii) analyze the population structure of the IAPAR collection of *C*. *arabica* genotypes encompassing wild accessions; (iii) perform a GWAS to decipher the genetic basis of lipid and diterpene contents within the broad-based Ethiopian collection; and (iv) draw consequences for coffee collections and *C*. *arabica* breeding programs.

## Results

### Lipid and diterpene profiles

The complete list of 107 accessions analyzed in the present study is shown in Supplementary Table S1. We observed a high variability among the accessions for all traits analyzed (Table 1). There was a negative correlation between cafestol (CAF) and kahweol (KAH) contents (r = −0.30, p-value < 0.005). KAH content showed a significant correlation with total lipid content (r = 0.29, p-value < 0.005), whereas CAF content showed no correlation with total lipids (r = 0.08, p-value > 0.005).

**Table 1:** Mean, standard deviation (SD), minimum, and maximum phenotypic values and Pearson’s correlation of diterpenes cafestol and kahweol (expressed in mg.100 g^−1^ DW), cafestol/kahweol ratio and total lipids (expressed in g.100 g^−1^ DW) across 107 *C*. *arabica* accessions.

Trait	Mean	SD	Min	Max	Correlations			
					Cafestol	Kahweol	Ratio	Lipids
Cafestol	830.53	204.8	299.61	1308.97				
Kahweol	768.45	253.2	182.7	1400.49	−0.3*			
Ratio	1.24	0.9	0.32	7.16	0.65*	−0.72*		
Lipids	14.74	1.26	10.72	17.15	0.08	0.29*	−0.22*	

Correlations significantly different from 0 (α < 0.005) are indicated with an asterisk.

### Genotyping-by-sequencing and SNP detection

Due to the lack of a *C*. *arabica* reference genome, we used the publicly available genome assembly of its ancestor *C*. *canephora*. This reference genome was used to map the GBS tags and perform the SNP calling. GBS libraries yielded approximately 48 million single-end reads. Those reads produced 6,210,920 tags, of which 20% were aligned to unique positions. A total of 6,696 SNPs was identified, with an average depth of 39×. The SNPs were filtered based on minor allele frequency (MAF > 0.05) and call rate (>0.80). Thereafter, the resulting SNPs were filtered based on their heterozygosity (Ho): SNPs with Ho >0.9 were discarded. Filtering based on Ho was performed in order to eliminate SNPs deriving from *C*. *arabica* homeologous genomic regions in which different alleles are fixed in the two subgenomes (CaCe vs CaCc)^23^. A final set of 2,587 SNPs were obtained and used for further population structure and genome wide association analysis for the lipids and diterpenes contents.

### Population structure of the collection

**Figure 1:**
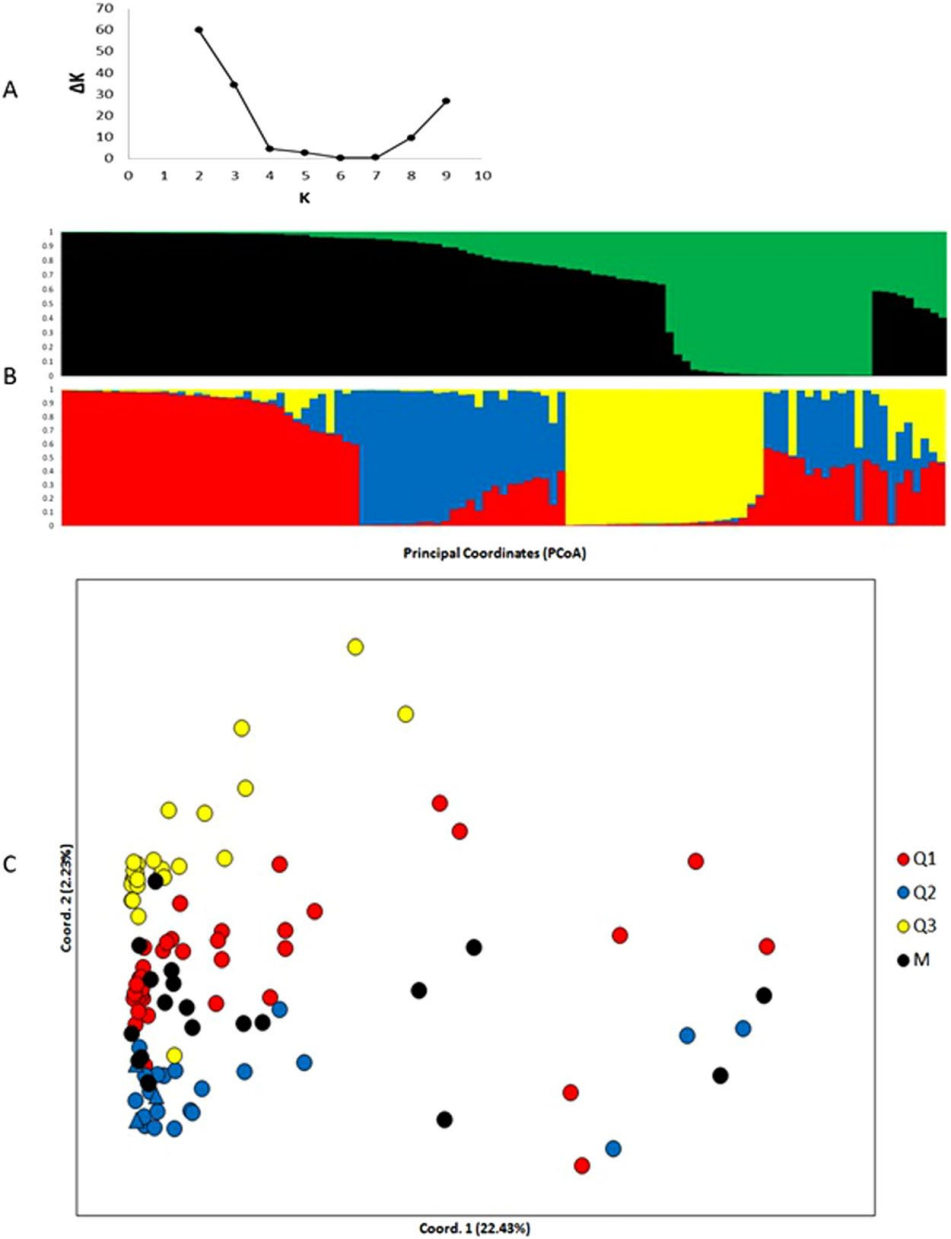
Population structure among 107 *Coffea arabica* accessions. (**A**) Evolution of ΔK values (y-axis) according to the number of genetic groups (x-axis), (**B**) barplot of the estimated membership coefficient (Q) of the 107 different accessions based on the 2,587 SNP for the K = 2 and K = 3, and (**C**) principal coordinate analysis (PCoA). In the PCoA individuals are coloured according to the STRUCTURE groups using K = 3. M group individuals are coloured in black.

Population structure analysis was performed using a Bayesian model-based approach implemented in STRUCTURE software (Fig. 1A). The STRUCTURE results based on three groups (K = 3) showed a high ΔK value, but the upper-most level of the structure was in two groups (K = 2) based on the Evanno criterion^24^.

The structure result using K = 2 (Fig. 1B) grouped all cultivars and accessions from the east side of the Great Rift Valley in the Q1 group (black). Meanwhile, the Q2 group (green) was exclusively composed of wild accessions from the west side of the Great Rift Valley. On the other hand, the structure result using K = 3 formed a Q1 group (red) composed of 37 genotypes from the west side of the Great Rift Valley. The Q2 group (blue) was formed by three traditional cultivars (Bourbon, Typica and Mundo Novo), five accessions from the east and 16 from the west side of the Great Rift Valley. The Q3 group (yellow) was composed of 25 genotypes, all wild accessions collected in the forests of western Ethiopia. The mixed group (M, individuals with admixture higher than 0.4) included nine accessions from the West side of the Great Rift Valley.

In a principal coordinate analysis (PCoA), the first two coordinates explained 25% of the total genetic variation (Fig. 1C). Similar to the STRUCTURE analysis, traditional cultivars were genetically closer to eastern Ethiopian genotypes than western Ethiopian genotypes.

The M group presented the highest intragroup diversity, showing an allele number average (Na), Shannon’s information index (I) and expected heterozygosity (He) mean of 1.97, 0.55, and 0.37, respectively (see Supplementary Table S2). This result can be explained by the fact that the M group is composed of mixed individuals. In the Q1, Q2, and Q3 groups, we observed 11, 15, and 6 private alleles, respectively. The M group did not contain private alleles. The most homogeneous and distant group in relation to the others was Q3, formed exclusively by wild accessions collected in forests of western Ethiopia.

**Figure 2:**
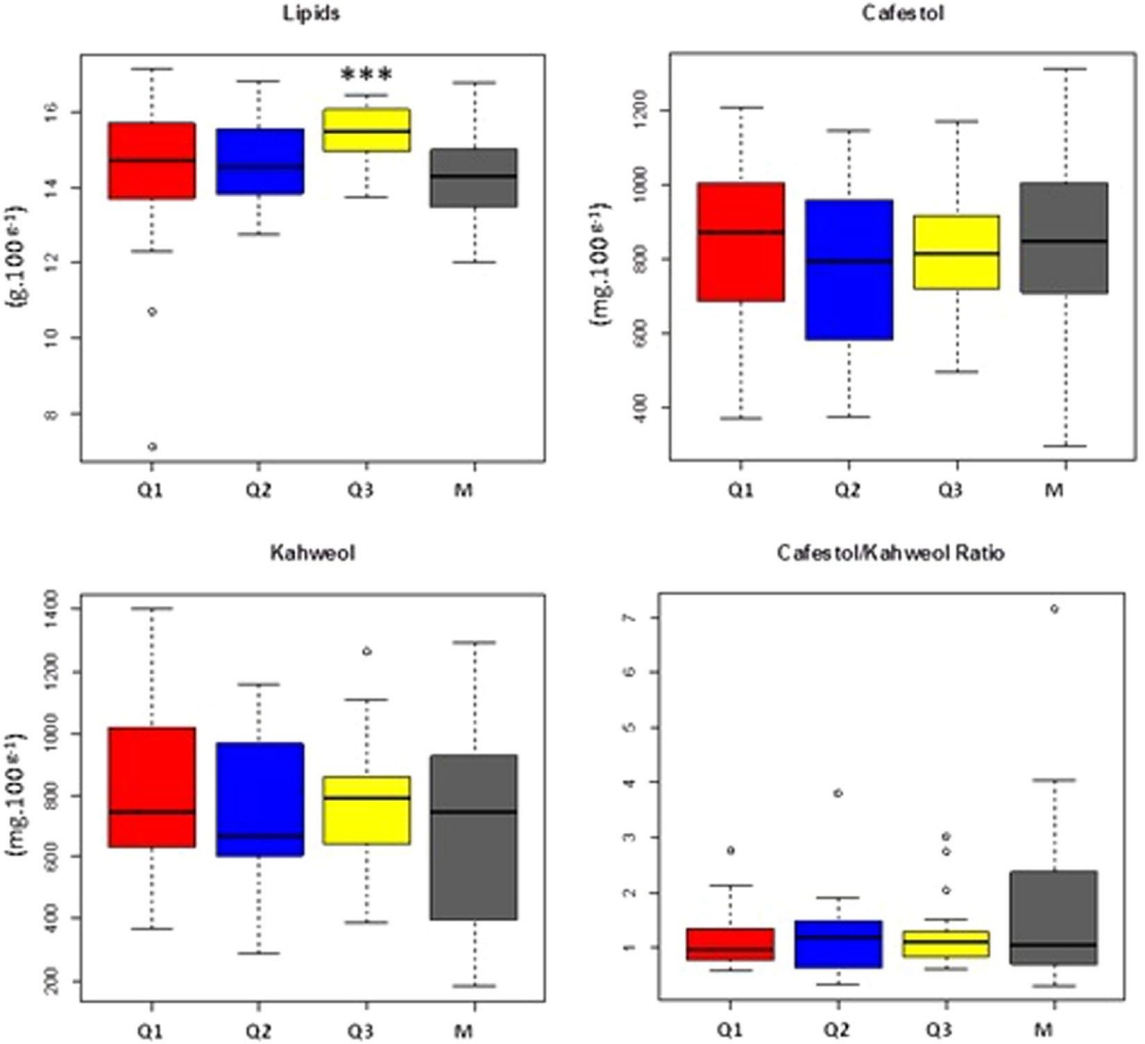
Lipid, cafestol, and kahweol contents and cafestol/kahweol ratio in the genetic groups identified by STRUCTURE analysis using K = 3. ***Significant by ANOVA (p = 0.05).

Comparing lipid, CAF, and KAH contents and CAF/KAH ratio among genetic groups (Fig. 2), we observed that the group composed of wild accessions (Q3) presented lower ranges of variation for all traits. In addition, according to ANOVA, Q3 had a higher lipid content than the other groups (p-value < 0.05). On the other hand, the M group presented a wide range of variation in all traits. The accessions with lower phenotypic values for all traits were sorted into the M group.

### Linkage disequilibrium analysis

The parameters r^2^ and r^2^_vs_ were estimated as a function of the physical distance between loci. We observed a linkage disequilibrium (r^2^_vs_, corrected for population structure and bias due to relatedness) decay below 0.2 at 185 Kbp (see Supplementary Fig. S1). Considering the values of r^2^ (uncorrected), we observe a linkage disequilibrium decay below r^2^ = 0.2 at 298 Kbp. With the r^2^_vs_ measure, lower values overall were obtained, as well as an expected exponential decline of linkage disequilibrium with distance, which demonstrated the efficiency of this measure in correcting bias. We also observed a difference between the estimated r^2^ and r^2^_vs_. The positive bias was removed across the whole chromosomal segment. However, for some close loci, the r^2^_vs_ estimate was larger than r^2^, leading to the removal of negative bias, as well. It is important to note that LD was calculated using the *C*. *canephora* ancestral genome as a reference, since there is no Arabica genome available.

### Genome-wide association mapping for lipids and diterpenes

To identify genomic regions associated with natural variation in lipids and diterpenes content in *C*. *arabica* beans, we performed GWAS using four different methods (mrMLM, ISIS EM-BLASSO, pLARmEB, and FASTmrEMMA) with 107 accessions. We identified a total of 21 SNPs associated with lipid (5), CAF (4), and KAH (3) contents and CAF/KAH ratio (9), which were distributed among all chromosomes (Table 2, and Supplementary Figures 1–4). Nine SNPs were associated with the traits analyzed by at least two methods. Two SNPs, one for CAF and one for KAH were identified by three methods (mrMLM, pLARmEB, ISIS EM-BLASSO). Using FASTmrEMMA method, no SNP was significantly associated. On the other hand, ISIS EM-BLASSO and pLARmEB were the methods identifying a high number of associated SNPs, 13 and 16 respectively.

**Table 2:** SNPs associated with lipid, cafestol, kahweol contents and cafestol/kahweol ratio detected by three different GWAS methods (mrMLM, ISIS EM-BLASSO, pLARmEB).

Trait	SNP	mrMLM(p-value)	ISIS EM-BLASSO(LOD value)	pLARmEB(p-value)
Lipid	S1_24382872			6.0 e-04
Lipid	S2_14041151			1.3 e-03
Lipid	S2_20725291			2.0 e-04
Lipid	S6_36332719		2.56	
Lipid	S8_25559761	3.27 e-05	3.56	
Cafestol	S3_7990620			4.0 e-04
Cafestol	S6_7853861		2.33	1.0 e-04
Cafestol	S11_29778697	1.77 e-06	3.64	1.49 e-06
Cafestol	S11_30776239		3.77	
Kahweol	S2_45775221	3.11 e-06	5.11	1.99 e-06
Kahweol	S4_5260584		3.1	
Kahweol	S8_17996908			9.0 e-04
Ratio	S2_15335083		4.83	2.73 e-05
Ratio	S2_15335417		2.24	2.73 e-05
Ratio	S2_48526210		7.7	8.02 e-09
Ratio	S4_3861777			7 e-04
Ratio	S5_27320598		2.18	
Ratio	S5_3863439			1.1 e-03
Ratio	S6_12529278	6.96 e-06		1.34 e-05
Ratio	S7_5138106		4.44	3.62 e-07
Ratio	S11_17488418		5.86	4.16 e-06

### Candidate genes co-localized with lipid- and diterpene-associated SNPs

For candidate gene mining, we considered only SNPs associated with traits that were detected by at least two methods. Remarkably, we found SNPs positioned within or near genomic regions coding for proteins involved in lipids and diterpenes metabolic pathways (Table 3).

**Table 3:** Candidate genes located in the vicinity of the SNPs presenting significant association with lipids, cafestol, kahweol contents, and with cafestol/kahweol ratio detected by at least two GWAS methods.

Trait	SNP	Candidate Gene	Distance (Kbp)	Functional Annotation
Lipid	S8_25559761	Cc08_g10680	48.8	Fatty acid desaturase (*FADS2*)
Cafestol	S6_7853861	Cc06_g09670	13.51	Flavin-containing monooxygenase (*FCM*)
Cafestol	S11_29778697	Cc11_g12750	1.42	Cytochrome P450 704 (*CYP704*)
Kahweol	S2_45775221	Cc02_g33380	22.74	Long chain acyl-CoA synthetase (*LCAS*)
Ratio	S2_15335417	Cc02_g16540	47.66	Triosephosphate isomerase (*TPIP1*)
Ratio	S2_48526210	Cc02_g34890	95.91	Dihydrolipoyl dehydrogenase (*lpdA*)
Ratio	S6_12529278	Cc06_g14660	36.99	Momilactone A synthase (*MAS*)
Ratio	S7_5138106	Cc07_g06960	6.06	Acyl-CoA N-acyltransferases (*NAT*)
Ratio	S11_17488418	Cc11_g04400	INSIDE	TATA-binding protein-associated factor 172 (*BTAF1*)

**Figure 3:**
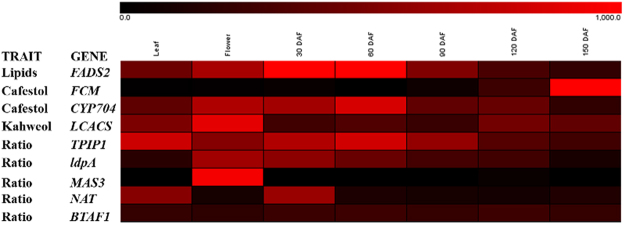
Heat map of digital gene expression patterns of nine genes co-localized with SNPs associated with lipids, CAF, KAH or Ratio (CAF/KAH) in coffee leaf, flower and fruit tissues (30 to 150 DAF). The intensity of the red color is proportional to the gene expression profile, and black color means low gene transcriptional activity, according to the RPKM values.

RNA-seq data obtained from coffee leaves, flowers and fruit tissues from 30 to 150 days after flowering (DAF) from a previous study^25^ were used to explore the gene expression patterns of some of the candidate genes identified (Fig. 3). Interestingly, with one exception (*BTAF1*), all the genes showed stronger expression profile in flowers and or fruit organs.

### Genomic signatures of selection among genetic groups

**Figure 4:**
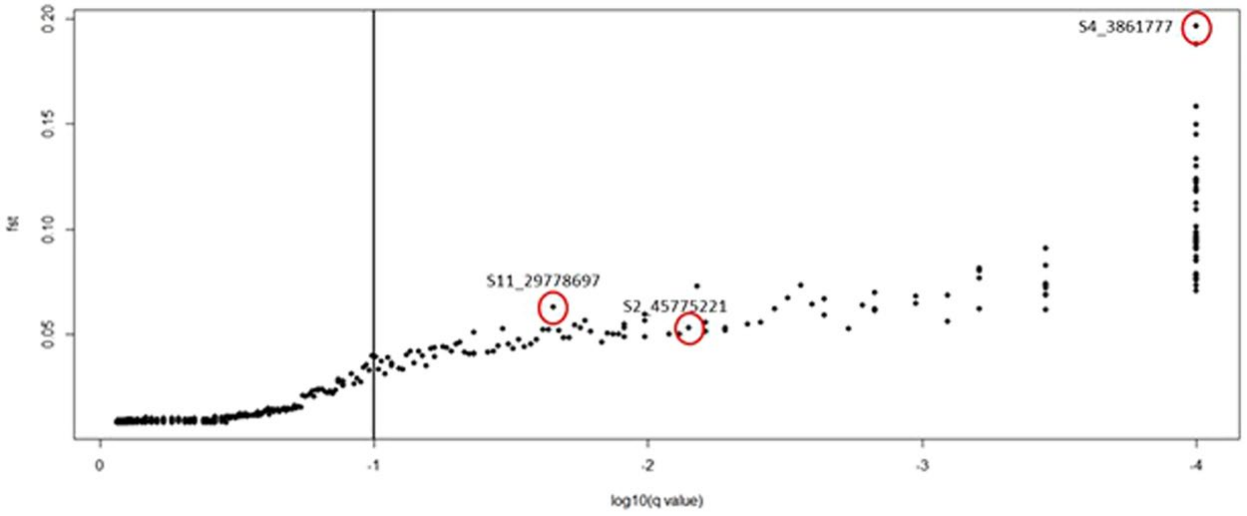
BAYESCAN results showing 139 SNPs under directional selection among STRUCTURE groups using K = 3 (FDR = 0.1). Red circles indicate trait-associated-SNPs detected through GWAS analysis.

Among 2,587 SNPs analysed, 139 present signature of diversifying selection among genetic groups (Q1, Q2, and Q3), according with BAYESCAN results (Fig. 4).

Three of these SNPs were also identified as being associated with some of the traits analyzed in the GWAS. The frequency of the alternative alleles of these loci in the Q3 group, formed by wild accessions and collected in the western forests of Ethiopia, was very low compared to the Q1 group, which was composed of domesticated accessions with intermediate levels of breeding (Table 4) and the Q2 group, which is composed of accessions with higher levels of breeding, including traditional cultivars Typica, Bourbon, and Mundo Novo.

**Table 4:** SNPs under directional selection among genetic groups detected by BAYESCAN (FDR = 0.05) and presenting significant association with the quantitative traits analyzed. Frequencies of the alternative alleles in each STRUCTURE group (K = 3).

SNP	Trait	Allele	Q1 frequency	Q2 frequency	Q3 frequency
S4_3861777	Ratio	G	0.71	0.85	0.02
S2_45775221	Kahweol	T	0.14	0.38	0.02
S11_29778697	Cafestol	T	0.13	0.27	0.02

## Discussion

### Phenotypic analysis

The 107 genotypes analyzed presented high phenotypic variability for the lipid, CAF and KAH contents and for the CAF/KAH ratio. Other studies also report high genetic diversity in *C*. *arabica* accessions from primary diversity centers for bean physical, organoleptic and biochemical qualities displaying high variability^2,13^. According to these studies, the influence of geographical origin on these traits was evident. Interestingly, in the present study a large influence of the geographic origin on CAF, KAH and lipid contents in the beans was also observed. Wild accessions collected in the forests of the west side of Great Rift Valley presented higher lipid contents than cultivars.

Although biochemical compounds related to beverage quality traits in coffee, including lipid and diterpene contents^2,7,12^, have been already described, this is the first large-scale study using an Arabica population that includes several wild accessions from Ethiopia. Accessions with different lipid and diterpene contents may serve as a source of alleles for the development of plants with desirable lipid and diterpene contents in the beans. Therefore, the results of the present study can contribute to coffee breeding to deliver high-quality coffee varieties according to the consumer market demands.

### Genotyping-by-sequencing and SNP detection

We used the diploid genome of *C*. *canephora*^19^ as a reference to find SNP markers in the *C*. *arabica* genome. The high degree of conservation between both genomes is well known^15,26^ and allowed us to map tags from genotyping-by-sequencing (GBS) data for SNP identification. We identified a total of 6,696 SNPs. Those SNPs were further filtered for MAF, call rate and heterozygosity, generating 2,587 high quality SNPs for population structure and genome-wide association analyses. One of the main difficulties of working with polyploids is distinguishing true SNPs segregating in the subgenomes from homologous SNPs representing fixed differences between both ancestral diploids subgenomes^23^. Therefore, SNPs corresponding to the differences between both subgenomes (heterozygosity = 1) were discarded and the SNPs selected represent true variability in *C*. *arabica*. The number of detected SNPs was relatively low. This can be explained by the low genetic diversity of the species, which has a recent origin^15^. In addition, we used just one subgenome as a reference, and the number of TAGs mapped was low (22%). However, in a recent similar study using GBS in *C*. *canephora*, only 32% of TAGs were mapped using the same *C*. *canephora* genome reference^27^.

### Genetic diversity and population structure

Despite the wide geographical range of Arabica coffee cultivation, the number of cultivars used is very small: mainly *C*. *arabica* var. Typica, *C*. *arabica* var. Bourbon, their mutants and hybrids^28^. The narrow genetic base of those cultivars^9^ has resulted in a crop with homogenous agronomic behaviors^15^, including high susceptibility to biotic and climatic stress^29^ representing a breeding challenge due environmental changes or market demands. The genetic diversity analysis using SNP markers revealed that the collection of *C*. *arabica* used in this study has a higher genetic diversity than traditional cultivars, consistent with the great phenotypic variability observed for the biochemical characterization previously reported^2,7,12^. In this context, our Ethiopian germplasm collection has been shown to be a valuable source of novel favorable biochemical characteristic-related alleles, which can be explored by breeding programs.

In the STRUCTURE analysis using K = 3, all cultivars and genotypes from the east side of the Great Rift Valley were sorted into the same group (Q2). Previous genotypic characterization of this collection using microsatellite markers showed a subdivision of these genotypes only into two groups, from the west and east sides of the Rift Valley^9,11^.

Interestingly, the Q3 group, formed by wild accessions, presented a high lipid content in comparison to the other groups. This result indicates that the Q3 group contains alleles conferring differentiated lipid content in beans. In Ethiopia, this wild gene pool has been potentially threatened by forest fragmentation and degradation and by introgressive hybridization with locally improved coffee varieties^30^.

Our results reinforce the importance of preserving the germplasm of *C*. *arabica* from the origin center (Ethiopia). Both forest fragmentation and forest degradation can have a negative impact on the genetic diversity of forest plant species through increased genetic drift, reduced gene flow, and alteration of mating patterns resulting in increased inbreeding^31,32^. In addition, the widespread planting since the 1970s of a restricted set of locally improved coffee varieties, mainly genotypes resistant to coffee berry disease, in the forest and its surroundings may result in the replacement of a part of the wild gene pool with a small number of domesticated alleles^33,34^. This can result in loss of genetic variation from the original gene pool and may even have negative fitness consequences for the original populations^35^. Overall, our results can help us to define which accessions are more important to preserve in order to have a good genetic representation of the FAO collection. The genetic diversity of plants from the western region demonstrated the importance of carefully preserving and exploring the accessions from this region in order to increase genetic variability, especially for coffee beverage quality^12^. It is important to observe that our work was performed only with a subset of the full FAO collection. Studies using the whole collection and or focusing in the genotypes from the Western side of Great Rift Valley would be of great value for increase our knowledge on the phenotypic and genotypic diversity of *C*. *arabica*.

### Genome-wide association study

Several studies relating quantitative trait loci (QTLs) to cup quality compounds have been performed on *C*. *canephora*^35^ and other *Coffea* species^36^, but none has been reported for *C*. *arabica*. We performed GWAS for lipids and CAF and KAH diterpenes in coffee beans using 104 accessions from the FAO Ethiopian collection and three cultivars. We used 2,587 high-quality SNPs and identified 21 SNP/trait associations.

A common feature of the MLM-based GWAS methods is the one-dimensional genome scan, performed by testing one marker at a time. However, such a model does not facilitate good estimates of marker effects because the model is never correct if a trait is indeed controlled by multiple loci, which is the case for most complex traits^37^. Another problem with the method is the issue of multiple test corrections for the threshold value of significance testing. The typical Bonferroni correction is often too conservative, so many important loci may not pass the stringent criterion of significance testing^37^. The mrMLM method was efficient to identify genomic regions associated with lipid and diterpenes concentrations in coffee green beans, combining an efficient control of false positives with high power, as described by the authors of this method^37^.

### Candidate genes co-localized with lipid-associated SNPs

Coffee bean lipids are composed mainly of triacylglycerols, sterols and tocopherols, the typical components found in all common edible vegetable oils^4^. Insights into the details of lipid biosynthesis and information on the genes and enzymes involved in this process may lead to innovative strategies to modify the fatty acid composition and increase seed oil content. In the present study, we identified one lipid-associated SNP (S8_25559761) co-localized with the *Cc08_g10680* gene, which encodes a fatty acid desaturase (*FAD2*). Desaturase enzymes regulate the unsaturation of fatty acids through the introduction of double bonds between defined carbons of the fatty acyl chain. Very interestingly, the difference of diterpenes CAF and KAH is just one unsaturated carbon^38^, therefore the potential role of *FAD2* in KAH formation should be further investigated. In *Arabidopsis thaliana*, *FAD2* has been shown to be important in the seed oil biosynthesis pathway^39^. This gene was identified as associated with lipid content in corn grains^40^ and brassica^41^.

### Candidate genes co-localized with diterpene-associated SNPs

All plant diterpenoids are derived from only two five-carbon (C5) isoprenoids, isopentenyl diphosphate (IPP), and dimethylallyl diphosphate (DMAPP), produced via the cytosolic mevalonate (MVA) and the plastidial 2-C-methyl-D-erythritol-4-phosphate (MEP) pathways^38^. Sequential condensation of these units by transferases yields a handful of central prenyl diphosphate intermediates in terpenoid biosynthesis. Diterpenoids originate predominantly from the MEP pathway.

KAH and CAF are exclusive diterpenes of the *Coffea* genus^7^. They have a very similar chemical structure with one double bond difference in the aromatic hydrocarbon composed by twenty carbons^38^. In contrast to other biochemical compounds, the total amount of diterpenes does not significantly change among cropping years and environments^2^, suggesting that the production of these compounds is under strong genetic control. Terpene diversification is driven by the machinery consisting TPSs and cytochrome P450-dependent monooxygenases (*CYP*) genes. The latter is important for modifying and diversifying the terpenoid scaffolds by redox modification^42^. We identified one SNP associated with CAF (S11_29778697) that was co-localized with the gene Cc11_g12750, which encodes a cytochrome P450 704 (*CYP704*). Several *P450* genes are involved in secondary metabolite biosynthesis, including terpenoids^43,44^. *CYP704* in rice was also shown to provide lipid monomers for the synthesis of anther cutin^45^. Another SNP associated with CAF is positioned close to a monooxygenase. Monooxygenase was described as being directly involved in plant terpene biosynthesis^46^.

The SNP S2_45775221 associated with KAH is co-localized with Cc02_g33380, which encodes a long chain acyl-CoA synthetase (*LACS*). LACS proteins occupy a critical position in the biosynthetic pathways of nearly all fatty acid-derived molecules^47^. LACS proteins esterify free fatty acids to acyl-CoAs, a key activation step that is necessary for the utilization of fatty acids by most lipid metabolic enzymes. LACS proteins initiate the process of fatty acid β-oxidation. In oilseeds, carbon reserves are stored as triacylglycerol (TAG). With the onset of germination, lipases release free fatty acids from the TAG molecules. LACS proteins activate the free fatty acids to acyl-CoAs that enter the β-oxidation pathway in the glyoxysomes of the germinating seedling. The enzymes of the β-oxidation cycle completely degrade fatty acids by the sequential removal of two-carbon units, which are released in the form of acetyl-CoA. The resulting acetyl-CoA pool is essential for the production of cellular energy (through the tricarboxylic acid cycle) and for synthesis of sugars and other carbon skeletons. LACS were also identified as being associated with lipid content in maize^40^ and brassica^48^.

Among SNPs associated with the CAF/KAH ratio, one is co-localized with the gene Cc06_g14660, which encodes a diterpene synthase (momilactone A synthase). Momilactone A is a diterpenoid secondary metabolite that is involved in the defense mechanism of the plant^49^. In rice, a dehydrogenase also has been suggested to be involved in momilactone biosynthesis^50^. The SNP S2_48526210 is co-localized with the gene Cc02_g34890, which encodes a dihydrolipoyl dehydrogenase (lpdA). LpdA encoding the E3 subunits of both the pyruvate dehydrogenase and 2-oxoglutarate dehydrogenase complexes^51^.

As already demonstrated in the phenotypic analysis, the CAF/KAH ratio is significantly correlated with lipid content, and this could explain why some SNPs associated with lipid content are also co-localized with genes related to lipid metabolism. In addition, the initial steps of CAF and KAH biosynthesis use acetyl-CoA as a substrate^38^. One SNP associated with CAF/KAH ratio (S7_5138106) is co-localized with the gene Cc07_g06960, which encodes an acyl-CoA N-acyltransferases (*NAT*). N-Acyltransferase catalyzes the transfer of an acyl group to a substrate. Members of the N-acyltransferase superfamily have a similar catalytic mechanism but vary in the types of acyl groups they transfer, including those of the three main nutrient substances, saccharides, lipids and proteins. These substances participate in a common metabolic pathway mediated by acetyl-CoA in the tricarboxylic acid cycle and oxidative phosphorylation reactions. Acyl lipids have various functions in plants, and the structures and properties of the acyl lipids vary greatly even though they are all derived from the same fatty acid and glycerolipid biosynthesis pathway. Some acyl lipids, including jasmonic acid, participate in signaling pathways. Acyl-CoA and acyl-CoA N-acyltransferase are involved in these metabolic pathways, including pyruvate dehydrogenase and pyruvate, and they are involved in the metabolism of sugars in the citric acid cycle and fatty acids and fat metabolism required for the synthesis of flavonoids and related polyketides for the elongation of fatty acids involved in sesquiterpenes, brassinosteroids, and membrane sterols^47^.

We identified a SNP associated with CAF/KAH ratio (S2_15335417) that co-localized with the Cc02_g16540 gene, which encodes a plastidial triosephosphate isomerase (*pdTPI*). After germination, seedling establishment requires a transition from heterotrophic to autotrophic growth to sustain plant growth once storage reserves are used. This likely involves multiple plastid biosynthetic pathways. In plants, triose phosphate isomerase (TPIP; EC 5.3.1.1) is involved in several metabolic pathways operating during this transition, including glycolysis, gluconeogenesis, and the Calvin cycle^52^. In *Arabidopsis*, a plastid isoform of triose phosphate isomerase (*pdTPIP*) plays a crucial role in the transition from heterotrophic to autotrophic growth^54^. A T-DNA insertion in *Arabidopsis thaliana pdTPIP* resulted in a fivefold reduction in transcription, reduced *TPIP* activity, and a severely stunted and chlorotic seedling that accumulated dihydroxyacetone phosphate (*DHAP*), glycerol, and glycerol-3-phosphate^53^.

We observed the transcription pattern of the genes co-localized with associated SNPs. With one exception (*BTAF1*), the transcriptional data strongly corroborates to diterpene biochemical profile reported for the same organs^7,25^. Diterpenes are present mainly in roots, flowers and accumulated in fruits during its development reaching a peak around 120 DAF^7^. In flowers the presence of CAF is predominant and it will be very interesting to study the role of the *MAS* in CAF formation. Meanwhile *FADS2*, *CYP704* and *TPIP1*, showed a transcription pattern similar to KAH accumulation during coffee fruit development. The role of *FCM*, strongly expressed in the final stages of fruit maturation, also can be very interestingly with a potential role in the final composition of lipids in coffee grains.

Among all trait-associated SNPs detected by GWAS, three showed strong signals of directional selection between genetic groups identified using STRUCTURE with K = 3 (S4_3861777, S2_45775221, and S11_29778697). The Q3 group (wild accessions) presented very low frequencies of the reference alleles at these loci when compared to the Q1 group and especially compared to the Q2 group, which is composed of cultivated accessions. These observations indicate that domestication and the breeding process of *C*. *arabica* may have changed allelic frequencies of these loci in order to modulate lipids and diterpenes content, possibly resulting in differentiated beverages. In addition, lipids and terpenes are known as chemical compounds related to plant defense against herbivory, response to abiotic stress and coffee flavor^1,54^, all of which can also be related to the Arabica domestication process.

In summary, these findings identify candidate genes representing potential targets for improving beverage quality in relation to lipids and diterpenes composition. The information reported here can be a starting point to obtain plants with desirable content of lipids, CAF, and KAH by incorporating molecular breeding techniques to the traditional programs. Our analyses also allowed assessing the population structure and genetic relationships among genotypes of a *C*. *arabica* germplasm collection originated from FAO surveys in the 1960’s. We identified a great allelic richness in the accessions of Ethiopia, especially in the West side of the Great Rift Valley. Trait-associated-SNPs identified by GWAS may be helpful to develop Markers Assisted Selection strategies aiming to improve the biochemical quality of the coffee beans.

## Methods

### Plant material

The complete list of 107 accessions analyzed in the present study is shown in Supplementary Table S1. The FAO Ethiopian *C*. *arabica* collection as well as cultivars from the Instituto Agronômico do Paraná (IAPAR) breeding program were cultivated at its experimental station in Londrina, Brazil (23°23′00″S and 51°11′30″W). The soil is a red dystrophic latosol, and the average rainfall and temperature are 1,500 mm/year and 21 °C, respectively. The FAO collection at IAPAR comes from open-pollinated seeds from the original collection at CATIE (Costa Rica) introduced in Brazil in 1976, and kindly transferred from the Instituto Agronômico de Campinas (IAC) to IAPAR. Fruits were harvested from 107 genotypes between May to July 2011 at full maturity. Cherries were manually selected in order to avoid immature and damaged seeds, which were washed and sun-dried until they contained 12% moisture. Coffee beans were processed (husk and parchment removal) and standardized in grade 16-sized sieves (6.5 mm); all defective beans were discarded.

### Phenotyping for lipid and diterpene contents

Coffee beans were frozen using liquid nitrogen to prevent compound oxidation in the matrix and ground (0.5 mm particles) in a disk mill (PERTEN 3600, Kungens Kurva, Sweden). The milled samples were stored in plastic bags and kept in a freezer (−18 °C) until analysis. The moisture content (oven set at 105 °C to constant weight) was also determined to express the results in terms of dry weight. Cafestol (CAF) and kahweol (KAH) were analyzed by direct extraction using saponification and cleanup in terc-butyl-methyl-ether and water^2^. The extracts were identified and quantified by HPLC at 220 and 290 nm for CAF and KAH, respectively. A reversed-phase Spherisorb ODS 1 column (250 mm × 4.6 mm id 5 mm) (Waters, Milford, USA) and an acetonitrile: water (55:45) mobile phase were used to separate the compounds. Quantification was carried out by external standardization, generating calibration curves with CAF and KAH content between 50 and 1,000 mg.100 g^−1^ (six different concentrations in triplicate). To determine the lipid content of ground coffee beans, the methods described in the Association of Official Analytical Chemists (AOAC)^55^ using petroleum ether as a solvent was employed.

### Genotyping-by-sequencing

DNA extractions were performed from leaves using a modified CTAB protocol^56^. GBS was performed by the Genomic Diversity Facility LIMS at Cornell University. The *PstI* restriction enzyme was used for library preparation^57^. Single-end sequencing of multiplexed GBS libraries were performed on Illumina HiSeq 2000 equipment, with 159 samples in two 96-well multiplex plates. Single nucleotide polymorphisms were identified using the TASSEL-GBS pipeline^58^ in TASSEL software version 3.0.166. Briefly, raw FASTQ sequences were trimmed to remove barcodes and reads from each of the four FASTQ files were collapsed into one master TagCounts file containing unique tags along with their associated read count information. Tags aligned to unique positions on the *C*. *canephora* reference genome^19^ were used for SNP calling. SNP discovery was performed for each set of tags that aligned to the exact same starting genomic position and strand. SNP genotyping was determined by the default binomial likelihood ratio method of quantitative SNP calling in TASSEL 3.0.166^58^. GBS SNP calling was performed using the *C*. *canephora* genome as reference. Quality control of the SNPs was performed using the parameters of call rate (CR > 80%), minor allele frequency (MAF > 5%), and heterozygosity (Ho < 0.9).

### Assessment of genetic diversity using SNP markers

According to the whole set of SNP, we estimated mean number of alleles (Na), percentage of polymorphic loci (P), expected heterozygosity (He), Shannon’s information index (I) and number of private alleles in each genetic group using GenAlEx 6 software^59^.

### Population structure analysis

We performed principal coordinate analyses (PCoAs) via covariance matrices with data standardization using GenAlEx 6 software to assess and visualize genetic relationships among genetic groups and individuals.

Genetic structure was estimated using the model-based Bayesian method implemented in STRUCTURE software version 2.3.4^60^. Allele frequencies of each K cluster (from 2 to 10) were estimated. We assumed a single domestication event and restricted our analysis to the correlated frequency model. We used a 10^5^ burn-in period and 10^5^ iterations, as these parameters resulted in relative stability of the results with 10 runs per K value. The genome composition (genome plot) of the accessions was represented for each K. Only accessions displaying a membership larger than 0.6 were assigned to a genetic group, resulting in assignments for 80% of the accessions. Accessions with memberships lower than 0.6 were assigned to a mixed cluster (M). We used the *ΔK* criterion^24^ in Structure Harvester software^61^ to estimate the upper-most level of structure.

### Linkage disequilibrium analysis

Pairwise linkage disequilibrium (LD) between SNP markers was calculated to evaluate the extent of LD decay. Only pairs of markers with distances at most 20 Mbp from each other were considered. LD was estimated using the parameter r^2^_vs_ obtained by considering the population structure and cryptic relatedness using the R package ‘LDcorSV’ version 1.3.1^62^. An identity-by-state (IBS) centered kinship matrix was calculated using TASSEL software version 5.2.20^63^. A population structure matrix (Q matrix) was obtained using STRUCTURE software version 2.3.4^61^ (K = 2).

### Genome-wide association mapping for lipids and diterpenes

To identify SNPs and candidate genes associated with natural variation in lipid and diterpene contents in Arabica beans, we performed GWAS using four methods: multi-locus random-SNP-effect mixed linear model (mrMLM), FAST multi-locus random-SNP-effect EMMA (FASTmrEMMA), integrative sure independence screening EM-Bayesian LASSO (ISIS EM-BLASSO), and polygenic-background-control-based least angle regression plus empirical Bayes (pLARmEB).

The mrMLM method used a random-SNP-effect MLM (RMLM) and a multi-locus RMLM (mrMLM) for GWAS. The mrMLM treats the SNP-effect as random, but it allows a modified Bonferroni correction to calculate the threshold p-value for significance tests. The mrMLM is a multi-locus model including markers selected from the RMLM method with a less stringent selection criterion. Due to the multi-locus nature, no multiple test correction is needed. The results from real data analyses and simulation studies show that the mrMLM has the highest power for quantitative trait nucleotide QTN detection, the best fit for genetic models, the minimal bias in the estimation of the QTN effect, and the strongest robustness, compared with the RMLM and the EMMA^37^. For the mrMLM method, the parameters used were critical p-value in rMLM = 0.01, search radius of candidate gene (Kb) = 20, critical LOD score in mrMLM = 3.

In the FASTmrEMMA method, a new matrix transformation is constructed to obtain a new genetic model that includes only QTN variation and normal residual error; allowing the number of nonzero eigenvalues to be one and fixing the polygenic-to-residual variance ratio is used to increase computing speed^65^. All the putative QTNs with the ≤0.005 p-values in the first step of the new method are included in one multi-locus model for true QTN detection. Owing to the multi-locus feature, the Bonferroni correction is replaced by a less stringent selection criterion. The results from analyses of both simulated and real data showed that FASTmrEMMA is more powerful in QTN detection, model fit and robustness, has less bias in QTN effect estimation, and requires less running time than the current single- and multi-locus methodologies for GWAS, such as E-BAYES, SUPER, EMMA, CMLM and ECMLM^64^. For FASTmrEMMA, we used the critical p-value in the first step of FASTmrEMMA = 0.005 and critical LOD score in the last step of FASTmrEMMA = 3^64^.

ISIS EM-BLASSO uses an iterative modified-sure independence screening (ISIS) approach in reducing the number of SNPs to a moderate size^65^. Expectation-maximization (EM)-Bayesian least absolute shrinkage and selection operator (BLASSO) is used to estimate all the selected SNP effects for true quantitative trait nucleotide (QTN) detection. Monte Carlo simulation studies validated this method, which has the highest empirical power in QTN detection and the highest accuracy in QTN effect estimation, and it is the fastest, compared to the efficient mixed-model association (EMMA), smoothly clipped absolute deviation (SCAD), fixed and random model circulating probability unification (FarmCPU), and multi-locus random-SNP-effect mixed linear model (mrMLM)^65^. For the ISIS EM-BLASSO method, we considered a critical p-value = 0.01.

The pLARmEB method integrates a least angle regression with empirical Bayes to perform multi-locus GWAS under polygenic background control^66^ using an algorithm of model transformation that whitened the covariance matrix of the polygenic matrix K and environmental noise. Markers on one chromosome are included simultaneously in a multi-locus model and least angle regression is used to select the most potentially associated single nucleotide polymorphisms (SNPs), whereas the markers on the other chromosomes are used to calculate a kinship matrix as a polygenic background control. The selected SNPs in the multi-locus model are further detected for their association with the trait by empirical Bayes and likelihood ratio test. The results from the simulation studies showed that pLARmEB was more powerful in QTN detection and more accurate in QTN effect estimation, had lower false positive rates and required less computing time than Bayesian hierarchical generalized linear model, efficient mixed model association (EMMA) and least angle regression plus empirical Bayes. For the pLARmEB method, the parameters used were critical LOD score = 2 and the number of potentially associated variables selected by LARS = 50.

All these analyses were performed using the mrMLM package^37^ in the R program. To control the effect of population structure, we used a Q matrix generated by STRUCTURE software considering K = 2. To control the bias generated by the kinship effects between individuals, an identity by state (IBS) kinship matrix was used. The Coffee Genome Hub database^20^ was used to identify *C*. *canephora* genes located in the interval of 100 Kbp encompassing significant SNPs.

The digital gene expression pattern was obtained using RPKM values from coffee leaves, flowers and fruit tissues from 30 to 150 days after flowering published in a previous study^25^. Graphic were developed using Genesis Software version 1.8.1^67^.

### Detection of SNPs under directional selection among genetic groups

To detect loci under directional selection among genetic groups identified using STRUCTURE analysis, we used the Bayesian approach of BAYESCAN 2.01^68^. BAYESCAN was run with burn-in = 50,000, thinning interval = 30, sample size = 5,000, number of pilot runs = 50, length of pilot runs = 5,000, and the false discovery rate (FDR) threshold 0.1.

## Electronic supplementary material

Supplementary Information
