# Supplementary material for: Genome-wide association study reveals candidate genes influencing lipids and diterpenes contents in Coffea arabica L
Source: Sci Rep. 2018 Jan 11;8:465. doi: 10.1038/s41598-017-18800-1 (PMC5764960; doi:10.1038/s41598-017-18800-1)
Supplement: Supplementary file 1 — Supplementary Information [file 41598_2017_18800_MOESM1_ESM.pdf]

Genome-wide association study reveals candidates genes influencing lipids and diterpenes contents in *Coffea arabica* L.

Gustavo C. Sant'Ana[1,2,3,6], Luiz F. P. Pereira[1,3*], David Pot[2,6], Suzana T. Ivamoto[1,4], Douglas S. Domingues[4], Rafaelle V. Ferreira[1], Natalia F. Pagiatto[1], Bruna S. R. Da Silva[1], Lívia M. Nogueira[1], Cintia S. G. Kitzberger[1], Maria B. S. Scholz[1], Fernanda F. De Oliveira[1], Gustavo H. Sera[1], Lilian Padilha[3], Jean-Pierre Labouisse[2,6], Romain Guyot[5], Pierre Charmetant[2,6], Thierry Leroy[2,6]

[1]Instituto Agronômico do Paraná, Laboratório de Biotecnologia Vegetal, 86047902, Londrina, PR, Brazil.
[2]CIRAD, UMR AGAP, F-34398 Montpellier, France.
[3]Empresa Brasileira de Pesquisa Agropecuária, 70770901, Brasília, DF, Brazil.
[4]Universidade Estadual Paulista, Instituto de Biociências, 13506900, Rio Claro, SP, Brazil.
[5]IRD, CIRAD, Univ. Montpellier, IPME, BP 64501, 34394, Montpellier, France
[6]AGAP, Univ. Montpellier, CIRAD, INRA, Montpellier SupAgro, Montpellier, France.

*Corresponding Author: filipe.pereira@embrapa.br

Supporting information

Additional supporting information may be found in the online version of this article.

Supplementary Table S1. List of 107 *Coffea arabica* accessions analyzed in this study.

Supplementary Table S2. Sample size (N), percentage of polymorphic loci (% P), allele number average (Na), Shannon's information index (I), expected heterozygosity (He), and number of private alleles in each genetic group identified by STRUCTURE analysis using K = 3.

Supplementary Figure S1. Linkage disequilibrium analysis of 107 *Coffea arabica* accessions.

Supplementary Figure S2. GWAS results for lipid content among 107 *Coffea arabica* accessions.

Supplementary Figure S3. GWAS results for cafestol content among 107 *Coffea arabica* accessions.

Supplementary Figure S4. GWAS results for kahweol content among 107 *Coffea arabica* accessions.

Supplementary Figure S5. GWAS results for cafestol/kahweol ratio among 107 *Coffea arabica* accessions.

Supplementary Table S1. List of 107 *Coffea arabica* accessions analyzed in this study

Genotypes	Side of Great Rift Valey	Local, Province	Coordenates	Sample Charaterization	STRUCTURE Group (K=3)
E046	West	Sedecha North of Jimma, Kaffa Jimma	7°57N 36°48E	Naturalized Forest	Q1
E047	West	Sedecha North of Jimma, Kaffa Jimma	7°57N 36°48E	Naturalized Forest	Q1
E055	West	Bada Buna, Kaffa Jimma	7°4N 38°52E	Naturalized	Q1
E057	West	Bada Buna, Kaffa Jimma	7°4N 38°52E	Naturalized	Q1
E061	West	Bada Buna, Kaffa Jimma	7°4N 38°52E	Naturalized	Q1
E068	West	Saredo, Kaffa Jimma	8°05N 36°53E	Farm under shade	Q1
E071	West	Sedecha North of Jimma, Kaffa Jimma	7°57N 36°48E	Naturalized Forest	Q1
E080	West	Cossa Kabenna, Kaffa Jimma	7°58 N 36°53E	Farm	Q1
E081	West	Norte de Ghembi, Kaffa Jimma	8°5N 36°54E	Naturalized Plants	Q1
E126	West	Gorei, Kaffa Jimma	6°42N 35°34E	NI	Q1
E130	West	Ota, Kaffa Jimma	7°51N 36°37E	Farm	Q1
E146	West	Ghera, Illubador	7°14N 36°24E	Farm open field	Q1
E152	West	Ghera, Illubador	7°14N 36°24E	Farm open field	Q1
E183	West	Between Agaro y Ghera, Kaffa Jimma	NI	Mix cultivated and naturalized	Q1
E254	West	Chochi, Kaffa Jimma	7°5N 36°45E	Wet processed	Q1
E279	West	22 km West of Bonga, Kaffa	7°15N 36°8E	Farm	Q1
E298	West	North of Ghembi, Kaffa Jimma	8°5N 36°54E	Naturalized	Q1
E308	West	Doyo, Kaffa Jimma	7°37N 36°46E	Farm open field	Q1
E315	West	Saredo, Kaffa Jimma	8°05N 36°53E	Farm under shade	Q1
E320	West	Saredo, Kaffa Jimma	8°05N 36°53E	Farm under shade	Q1
E335	West	Ghera, Kaffa Jimma	7°14N 36°24E	Farm open field	Q1
E368	West	Trail to Afallo, Illubador	7°45N 36°18E	NI	Q1
E370	West	Trail to Afallo, Illubador	7°45N 36°18E	NI	Q1
E386	West	5 km North of Afallo, Kaffa Jimma	7°43N 36°18E	Naturalized	Q1
E389	West	5 km North of Afallo, Kaffa Jimma	7°43N 36°18E	Naturalized	Q1
E401	West	Ota, Kaffa Jimma	7°51N 36°37E	Farm	Q1
E404	West	Ota, Kaffa Jimma	7°51N 36°37E	Farm	Q1
E464	West	7 km Southeast Teppi, Illubabor	NI	Forest	Q1
E467	West	10 Km southwest Agaro, Fichi Village, Kaffa Jimma	7°39N 36°33E	Farm	Q1
E478	West	Norte de Ghembi, Kaffa Jimma	8°5N 36°54E	Naturalized Plants	Q1
E481	West	Norte de Ghembi, Kaffa Jimma	8°5N 36°54E	Naturalized Plants	Q1
E490	West	Norte de Ghembi, Kaffa Jimma	8°5N 36°54E	Naturalized Plants	Q1
E494	West	Cossa Kabenna, Kaffa Jimma	7°58 N 36°53E	Farm	Q1
E340	West	Ghera, Kaffa Jimma	7°14N 36°24E	Farm open field	Q1
E025	East	NI	NI	NI	Q1
SEL106	NI	NI	NI	NI	Q1
E017	East	Wanago, Sidamo	6°20N 38°15E	Wet processed	Q2
E018	East	Yirga Cheffe, Sidamo	6°12N 38°13E	Farm under shade	Q2
E021	East	Camino a Soddu, Sidamo	7°8N 37°58E	Farm open field	Q2
E037	East	Wondo Genet, Shoa	7°7N 38°40E	Farm open field	Q2

E237	East	Between Yirgalem y Bira, Sidamo	NI	Farm	Q2
E039	West	Chochi, Kaffa Jimma	7°50N 36°45E	Wet processed	Q2
E085	West	Trail Gera to Afallo, Illubador	7°45N 36°18E	Farm open field	Q2
E174	West	SouthWest of Suntu, Kaffa Jimma	8°8N 36°53E	Farm	Q2
E213	West	Teppi, Illubabor	7°9N 35°18E	Forest Mix	Q2
E261	West	Doyo, Kaffa Jimma	7°37N 36°46E	Farm open field	Q2
E265	West	Balt, Kaffa Jimma	NI	Shade	Q2
E267	West	Balt, Kaffa Jimma	NI	Shade	Q2
E270	West	Kursi in Chersi, Kaffa Jimma	6°10N 30°33E	Shade	Q2
E283	West	Wush Wush, Kaffa Jimma	7°15N 36°8E	Farm	Q2
E302	West	Doyo, Kaffa Jimma	7°37N 36°46E	Farm open field	Q2
E327	West	Ghera, Kaffa Jimma	7°14N 36°24E	Farm open field	Q2
E332	West	Ghera, Kaffa Jimma	7°14N 36°24E	Farm open field	Q2
E383	West	Afallo trail; Kaffa Jimma	7°43N 36°18E	Trail	Q2
E408	West	Between Bonga and Wush Wush, Kaffa Jimma	7°15N 36°13E	Wet processed	Q2
E409	West	Between Bonga and Wush Wush, Kaffa Jimma	7°15N 36°13E	Wet processed	Q2
E419	West	Bonga, Kaffa Jimma	7°15N 36°13E	Cultivated	Q2
M7846	West	NI	NI	Cultivated	Q2
Bourbon		NI	NI	Cultivated	Q2
M_Novo		NI	NI	Cultivated	Q2
Typica		NI	NI	Cultivated	Q2
E114	West	Mizan Tefari, Kaffa Jimma	6°55N 35°25E	Farm open field	Q3
E116	West	Ainamba, Kaffa Jimma	6°35N 35°28E	Naturalized Under tree	Q3
E118	West	Korcha, Illubabor	7°8N 35°24E	Rain forest	Q3
E124	West	Geisha, Kaffa Jimma	6°38N 35°30E	Forest	Q3
E189	West	Teppi, Illubabor	7°9N 35°18E	Forest Mix	Q3
E199	West	Teppi, Illubabor	7°9N 35°18E	Forest Mix	Q3
E201	West	Teppi, Illubabor	7°9N 35°18E	Forest Mix	Q3
E208	West	Teppi, Illubabor	7°9N 35°18E	Forest Mix	Q3
E209	West	Teppi, Illubabor	7°9N 35°18E	Forest Mix	Q3
E218	West	Teppi, Illubabor	7°9N 35°18E	Forest Mix	Q3
E220	West	Teppi, Illubabor	7°9N 35°18E	Forest Mix	Q3
E325	West	Ghera, Kaffa Jimma	7°14N 36°24E	farm open field	Q3
E439	West	8 km East of Teppi, Illubabor	7°10N 35°18E	Forest	Q3
E450	West	8 km East of Teppi, Illubabor	7°10N 35°18E	Forest	Q3
E454	West	10 km Southeast Teppi, Illubabor	7°9N 35°22E	Forest	Q3
E456	West	10 km Southeast Teppi, Illubabor	7°9N 35°22E	Forest	Q3
E457	West	10 km Southeast Teppi, Illubabor	7°9N 35°22E	Forest	Q3
E458	West	10 km Southeast Teppi, Illubabor	7°9N 35°22E	Forest	Q3
E466	West	7 km Southeast Teppi, Illubabor	NI	Forest	Q3
E511	West	Ainamba, Kaffa Jimma	6°53N 35°28E	Naturalized	Q3
E514	West	Ainamba, Kaffa Jimma	6°53N 35°28E	Naturalized	Q3
E516	West	Tunteta, Kaffa Jimma	6°52N 35°27E	Cultivated	Q3
E571	West	Zeghie, Gojjam	11°42N 37°20	Cultivated in Forest	Q3

E621	West	Tunteta, Kaffa Jimma	6°52N 35°27E	Cultivated	Q3
E238	East	Aleta Wondo Colla, Sidamo	NI	Farm	M
E041	West	49 Km North of Jimma, Kaffa Jimma	7°57N 36°48E	Naturalized	M
E044	West	49 Km North of Jimma, Kaffa Jimma	7°57N 36°48E	Naturalized	M
E087	West	Edge Gera Village, Kaffa Jimma	7°14N 36°24E	Farm open field	M
E123a	West	Beru, Kaffa Jimma	6°15N 35°14	Forest	M
E123b	West	Beru, Kaffa Jimma	6°15N 35°14	Forest	M
E131	West	Ota, Kaffa Jimma	7°51N 36°37E	Farm	M
E148	West	Ghera, Illubador	7°14N 36°24E	Farm open field	M
E159	West	Ghera, Illubador	7°14N 36°24E	Farm open field	M
E221	West	Cerca de Teppi, Illubabor	7°9N 35°18E	Forest Mix	M
E268	West	Trail to Afallo, Illubador	7°45N 36°18E	Shade	M
E272	West	Kolu, Kaffa Jimma	6°95N 35°18E	NI	M
E287	West	Shebe, Kaffa Jimma	NI	NI	M
E301	West	Doyo, Kaffa Jimma	7°37N 36°46E	Farm open field	M
E324	West	Suntu, Kaffa Jimma	NI	NI	M
E326	West	Ghera, Kaffa Jimma	7°14N 36°24E	Farm open field	M
E331	West	Ghera, Kaffa Jimma	7°14N 36°24E	Farm open field	M
E364	West	Trail to Afallo, Illubador	7°45N 36°18E	Trail	M
E486	West	18 Km North of Ghembi, Kaffa Jimma	8°5N 36°54E	Naturalized Plants	M
E546	West	Wush Wush, Kaffa Jimma	7°15N 36°8	Farm	M
E552	West	Wush Wush, Kaffa Jimma	7°15N 36°8	Farm	M
E565	West	Zeghie, Gojjam	11°42N 37°20	Cultivated in Forest	M

Supplementary Table S2. Sample size (N), percentage of polymorphic loci (% P), allele number average (Na), Shannon's information index (I), expected heterozygosity (He), and number of private alleles in each group identified by STRUCTURE analysis considering K = 3

Group	N	% P	Na	I	He	Private alleles
Q1	36	97.37	1.97	0.53	0.36	11
Q2	25	97.30	1.97	0.54	0.37	15
Q3	24	93.36	1.93	0.51	0.35	6
M	22	99.34	1.99	0.55	0.37	0
Total	107	96.85	1.97	0.53	0.36	32

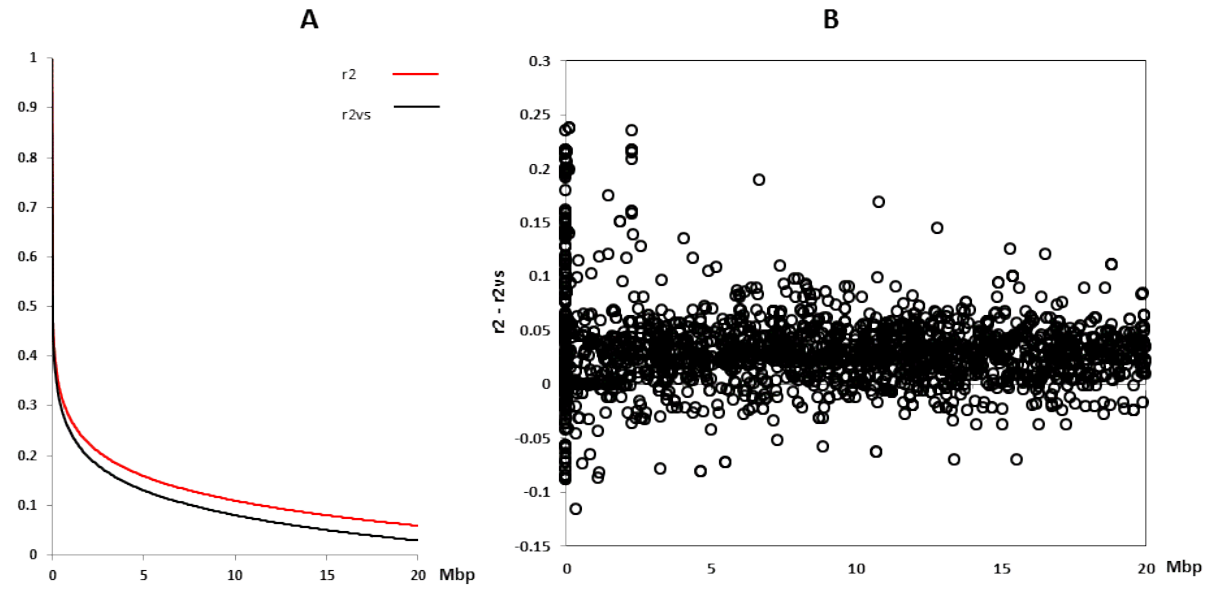

Supplementary Figure S1. Linkage disequilibrium analysis of 107 *Coffea arabica* accessions. (A) LD decay (measured by r^2 and r^2_{vs}) as a function of genetic distance between SNPs markers, and (B) difference between estimated r^2 and r^2_{vs} as a function of physical distance between pairs of loci (in Mbp).

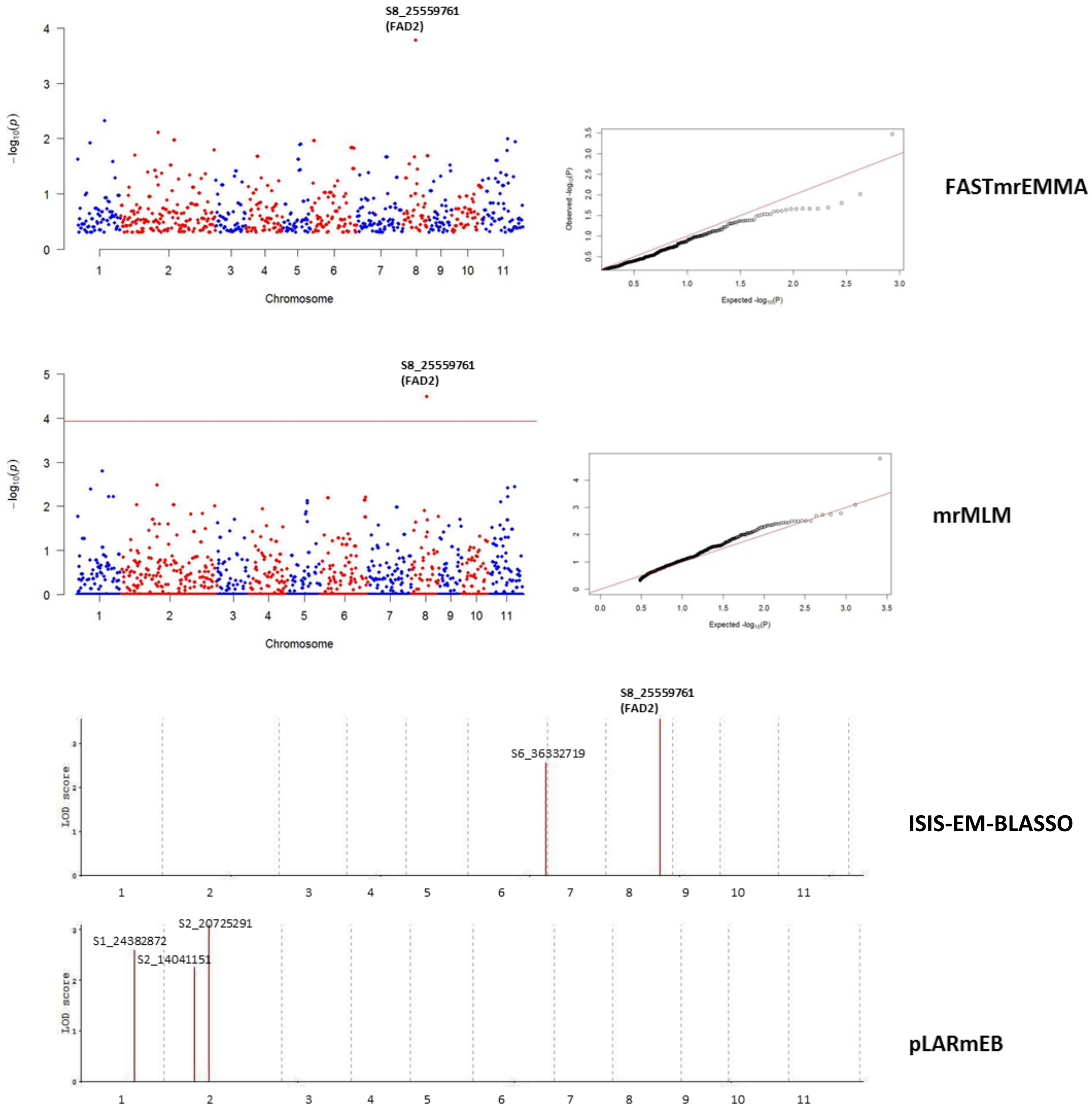

Supplementary Figure S2 – Manhattan plots and Q-Q plots showing GWAS results for lipid beans content among 107 *Coffea arabica* accessions using mrMLM and FASTmrEMMA methods, and LOD score plots representing results using ISIS-EM-BLASSO and pLARmEB methods.

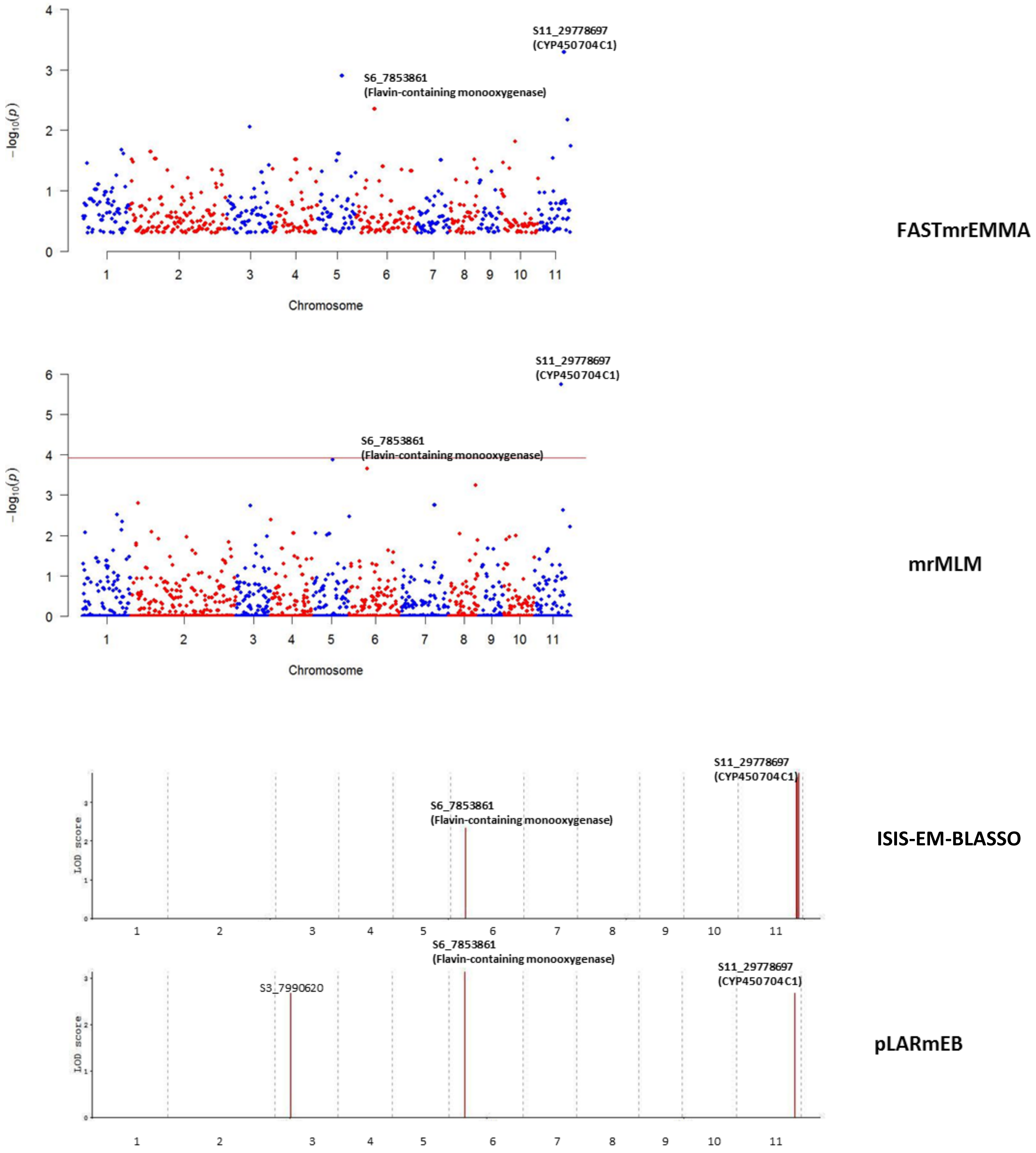

Supplementary Figure S3. Manhattan plots and Q-Q plots showing GWAS results for cafestol beans content among 107 *Coffea arabica* accessions using mrMLM and FASTmrEMMA methods, and LOD score plots representing results using ISIS-EM-BLASSO and pLARmEB methods.

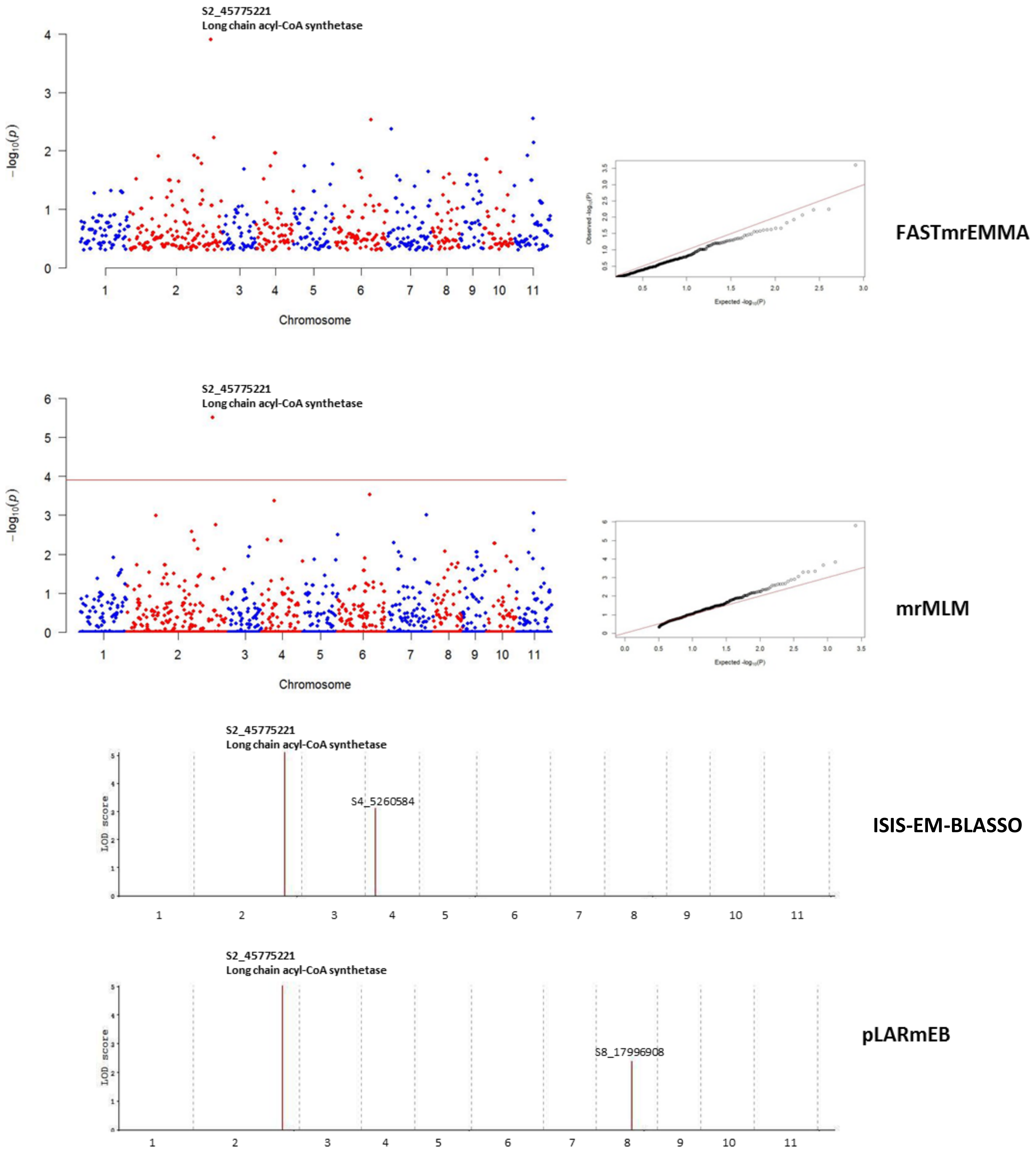

Supplementary Figure S4. Manhattan plots and Q-Q plots showing GWAS results for kahweol beans content among 107 *Coffea arabica* accessions using mrMLM and FASTmrEMMA methods, and LOD score plots representing results using ISIS-EM-BLASSO and pLARmEB methods.

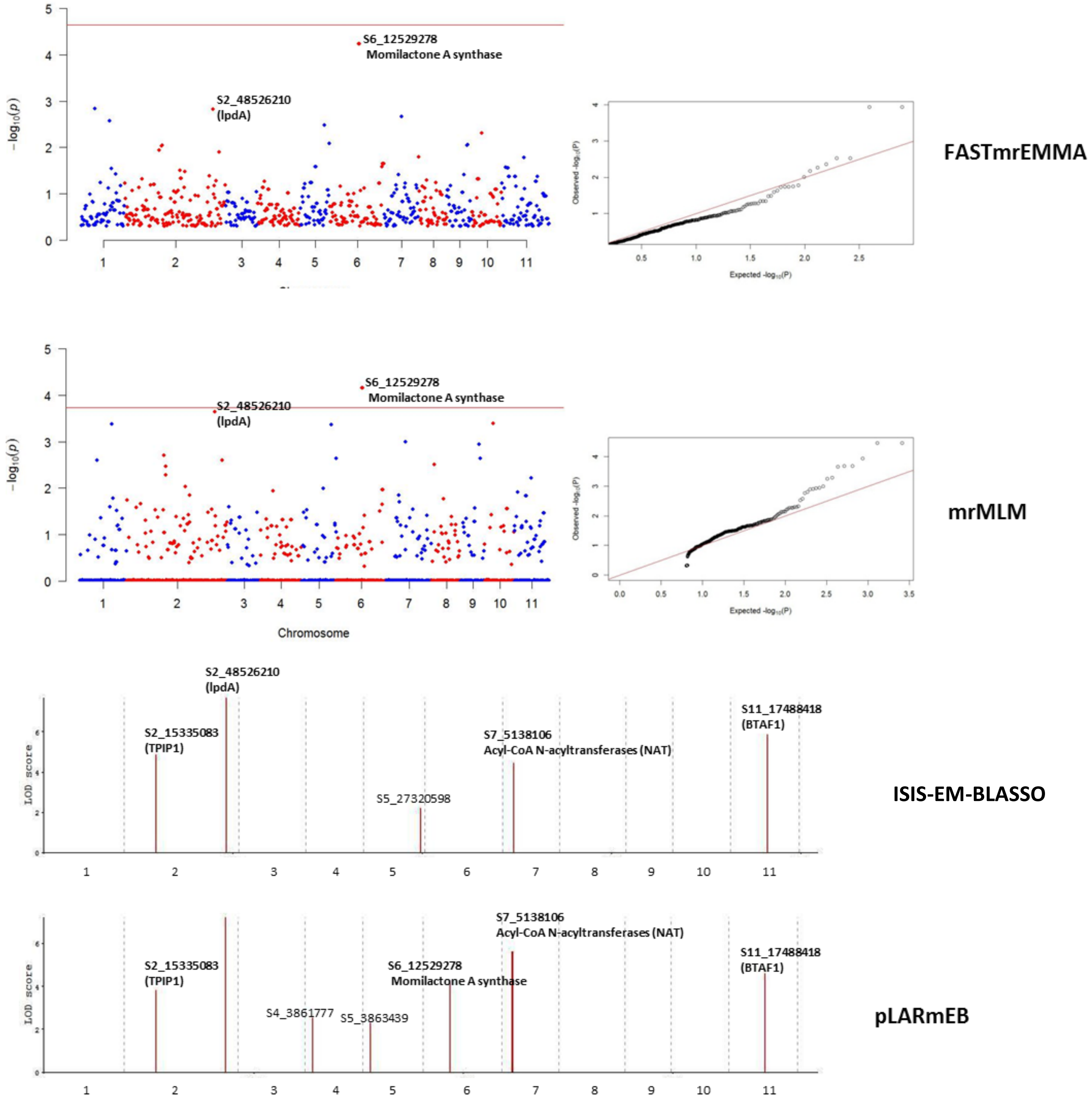

Supplementary Figure S5. Manhattan plots and Q-Q plots showing GWAS results for cafestol/kahweol ratio among 107 *Coffea arabica* accessions using mrMLM and FASTmrEMMA methods, and LOD score plots representing results using ISIS-EM-BLASSO and pLARmEB methods.
